# Making the Case for Joint Decision‐Making in Future Multipurpose Prevention Technology (MPT) Choice: Qualitative Findings on MPT Attribute Preferences from the CUPID Study (MTN‐045)

**DOI:** 10.1002/jia2.26024

**Published:** 2022-10-17

**Authors:** Nivedita L. Bhushan, Petina Musara, Miriam Hartmann, Marie C. D. Stoner, Shweta R. Shah, Josephine Nabukeera, Ivan Rukundo, Prisca Mutero, Megan A. Lewis, Jeanna Piper, Mary Kate Shapley‐Quinn, Juliane Etima, Alexandra M. Minnis

**Affiliations:** ^1^ RTI International Research Triangle Park North Carolina USA; ^2^ Clinical Trials Research Centre University of Zimbabwe Harare Zimbabwe; ^3^ Makerere University ‐ Johns Hopkins University Research Collaboration (MU‐JHU) Kampala Uganda; ^4^ National Institute of Allergy and Infectious Diseases National Institutes of Health Bethesda Maryland USA

**Keywords:** couples, multipurpose prevention technologies, HIV, contraception, pregnancy, decision‐making

## Abstract

**Introduction:**

Young women in sub‐Saharan Africa account for two‐thirds of all new HIV infections and face high rates of unintended pregnancy. Multipurpose prevention technologies (MPTs) are promising products under development that are designed to simultaneously prevent HIV and unintended pregnancy. Since MPTs will be used in the context of sexual relationships, ensuring acceptability and use requires understanding the role of male partners in MPT use decision‐making.

**Methods:**

This paper draws on qualitative data from 39 couples enrolled in the Microbicide Trials Network (MTN) 045 study, conducted in 2019–2020. Partners completed a discrete choice experiment (DCE), first separately and then jointly, to measure preferences for future MPT attributes and then completed a qualitative interview. We also draw on quantitative data from interviewer observation about who dominated the decision‐making process during the joint DCE. Content analysis was used to examine (1) how couples made decisions on existing non‐MPT HIV and pregnancy prevention products; (2) how couples made decisions on future ideal‐MPT product during the DCE; and (3) how these decision‐making processes varied by decision‐making dominance (10 male, 10 female and 19 equal) and interview type (19 joint and 20 separate).

**Results:**

Existing non‐MPT product decisions focused on trust between partners and product attributes, while future ideal‐MPT product decisions exclusively focused on product attributes. Across existing and future products, preferences for product attributes varied by gender. Male partners were most concerned with limiting side effects impacting sexual pleasure, female partners were most concerned with limiting side effects causing physical symptoms and both were concerned with the return to fertility. Across all dominance and interview types, couples reported making decisions together and female partners were often able to negotiate with male partners for their preferred product or set of product attributes.

**Conclusions:**

Research activities in this study provided an opportunity for couples to openly present their product attribute preferences to their partner, learn about their partner's attribute preferences, negotiate for their ideal set of attributes and ultimately choose attributes that benefited the couple without disempowering the female partner. Future research should focus on the utility of couple‐based decision‐making aids or similar tools for facilitating joint MPT decision‐making.

## 1 INTRODUCTION

In sub‐Saharan Africa (SSA), young women account for two‐thirds of new HIV infections and face high rates of unintended pregnancy [1, 2, 3, 4, 5, 6]. Dual method use, or the use of a condom and another contraceptive method, is the most reliable protection against sexually transmitted infections, such as HIV, and unintended pregnancy [7, 8, 9, 10]. Yet, the uptake of dual method use among women in SSA remains low [11, 12, 13, 14, 15, 16, 17]. Clinical studies, acceptability studies and market research suggest women would prefer a single product that prevents both HIV and unintended pregnancy [18, 19, 20, 21]. Currently, the only available single product, or modern multipurpose prevention technology (MPT), is the condom. However, condoms are not an ideal MPT because condom use must be negotiated between partners, often requires a male partner's acceptance, and has a high failure rate compared to other contraceptive methods [22, 23].

Several MPT products are in development, such as oral pills, vaginally inserted products (e.g. rings, inserts and films) and implants [24, 25]. Given that MPT products will be used in the context of sexual relationships, ensuring their acceptability and use requires understanding barriers and facilitators beyond individual and structural factors, such as interpersonal dynamics. Past HIV prevention trials of oral pre‐exposure prophylaxis (PrEP) and the dapivirine vaginal ring have shown the influence of male partners on product use and suggest the importance of their involvement and support in HIV and pregnancy prevention decisions [26, 27, 28, 29, 30, 31]. Previous studies have found that both genders are more likely to report using contraception if they have discussed family planning with their partners, even in couples with discordant fertility goals and where the male partner has a stronger preference for more children [32, 33, 34, 35, 36, 37]. In Malawi, increased contraceptive communication with male partners was a pathway through which a small‐group intervention increased contraceptive use in young women [38]. Despite evidence that HIV and pregnancy prevention communication increases method uptake and adherence, couple‐based communication about these topics is infrequent in SSA [39, 40]. Researchers attribute low levels of communication to prevailing gender norms that emphasize male‐dominated decision‐making regarding sexual and reproductive health issues [32, 39, 41].

This evidence resonates with Lewis's model of interdependence and communal coping [42]. The model posits that when couples are faced with a health decision, their predisposing characteristics (e.g. communication patterns, gender norms, trust and social support) influence a “transformation of motivation” otherwise known as their ability to reorient from individual‐centred decision‐making to couple‐centred decision‐making. Positive and equitable predisposing characteristics can lead to a transformation of motivation and in turn, allows a couple to engage in communal coping where they cooperatively engage in health‐enhancing behaviours (e.g. MPT use) [43, 44, 45, 46, 47]. Given the importance of male partner involvement for many women and the potential for more open communication in relationships to increase HIV prevention and contraceptive use, facilitating equitable decision‐making processes that lead to the transformation of motivation among couples constitutes a promising strategy to support future MPT use.

The Microbicide Trials Network (MTN) 045 study aimed to evaluate couples’ preferences for MPT product attributes and to understand the decision‐making process of couples’ when selecting an ideal MPT product. In the present analysis, we utilized qualitative data from the study to explore (1) how couples made decisions on existing HIV and pregnancy prevention products; (2) how couples made decisions on a future ideal MPT product; and (3) how these decision‐making processes varied by relationship dominance (male partner dominant, female partner dominant or equal contributions) and interview type (together as a couple or separate). By comparing decision‐making processes across existing products, future ideal products, gender dominance and interview type, we aim to understand couples’ priorities for a future MPT product and to inform the development of strategies to increase uptake and adherence to MPT products among couples when they become available for widespread use.

## 2 METHODS

### 2.1 Study overview

#### 2.1.1 Main study

**Figure 1:**
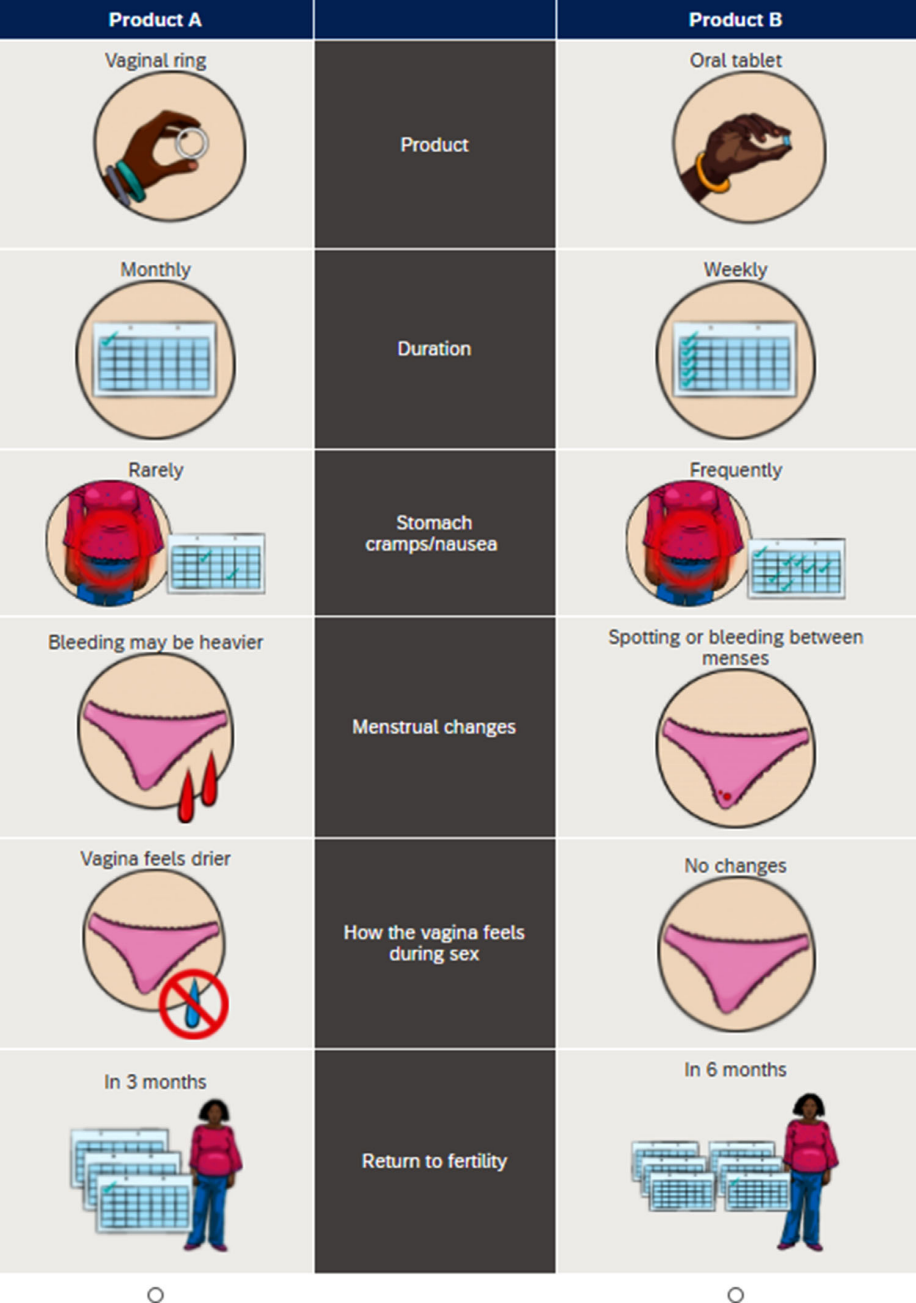
Example of a DCE choice set question. Participants were asked to select which MPT product they would want to use (females) or want their partner to use (males) for HIV and pregnancy prevention.

The MTN 045 study was designed to elicit couples’ preferences for MPT products in development and how those factors might affect acceptability and adherence to future MPT products [48]. The study enrolled 400 heterosexual couples in Uganda (*N* = 200) and Zimbabwe (*N* = 200) between November 2019 and December 2020. Eligible couples had been together for at least 3 months, were willing and able to provide consent, and expressed interest in contraception and/or HIV prevention. The female partner was required to be between the ages of 18 and 40 at enrolment and HIV negative by self‐report. The male partner was required to be 18 years or older. Participants were recruited from communities in Uganda and Zimbabwe through community meetings, stakeholder outreach, and community advisory board engagement activities. All participants were shown placebo versions of hypothetical product forms, and a video introducing the attributes of the hypothetical product forms, included in the discrete choice experiment (DCE). The DCE involved participants making nine choices between two hypothetical products (see Figure 1 for an illustrative example). Following each choice question, participants were asked their preference between their chosen product and male condoms. All participants completed an individual DCE and a joint DCE with their partner.

While the couple was completing the joint DCE, interviewers used a couple observation tool to capture couple dynamics. The tool was developed based on principles from psychology and couples counselling interventions [49, 50]. It included a checklist of attributes discussed and who dominated those discussions (male, female and both equally). Interviewers underwent interactive training on the use of the tool which included practising based on videos of couple interactions, the role of self‐awareness in observations and regular coaching in team meetings throughout the study. A minimum of 20% of sessions were jointly observed by two interviewers and inter‐observer reliability was calculated and monitored. Assessments on which observers disagreed were discussed and a consensus decision was reached. Across sites, the average agreement was 92% (standard deviation [SD] 0.07). Agreement was, on average, 94% (SD 0.05) in Zimbabwe and 86% (SD 0.11) in Uganda. Full details of recruitment procedures, DCE development and DCE results are detailed in the parent study publication [51].

#### 2.1.2 Present study

This analysis includes a subsample of 39 couples from the main study who were purposively subsampled for in‐depth qualitative interviews after both partners individually and jointly completed a DCE and data were collected through the couple observation tool on dominance. The research team at each site invited up to 20 couples to complete interviews, with interviews conducted either with both members of the couple together or separately. Interview questions and themes are detailed in Table 1. The team targeted roughly equal numbers of IDIs conducted with couples together or separately and as well as with couples representing a range of decision‐making dominance (e.g. male partner dominant, female partner dominant or equal contributions). In terms of communication dominance during the DCE, our qualitative sample included 19 couples observed as equal contributors, 10 couples that were male dominant and 10 couples that were female dominant. Couples were interviewed jointly (*N* = 19) or separately (*N* = 20), resulting in a total of 59 interviews (19 joint interviews and 40 individual interviews) Additional allowance for couples deemed as interesting cases was made for sample selection. IDIs took place at a separate visit that occurred within 1 month following the main study visit. All participants provided written informed consent at study enrolment and confirmed consent verbally prior to beginning the qualitative interview.

**Table 1:** Interview questions

Theme	Interview questions and illustrative probes
Relationship characteristics	Let us start by talking about your relationship. First, tell me how and where you met. ▪ What brought you two together? How long ago was that? ▪ What is changed in your relationship since then?
	How would you describe your relationship with other people now? ▪ What is your communication like? ▪ How do you express your feelings or opinions to each other? ▪ What are typical things you agree and/or disagree on?
	What is it like for couples in your community to talk about (1) pregnancy—including preventing, spacing or planning pregnancy and (2) HIV prevention? ▪ How open are couples with each other when discussing pregnancy/HIV prevention? How often do these conversations occur? ▪ What are some of the reasons why couples in the community may not talk about pregnancy prevention/HIV prevention with each other?
	Could you tell me about the last conversation you had about pregnancy prevention and HIV prevention with your partner? ▪ How do conversations about these topics come up? Under what circumstances? ▪ How often do you discuss these topics together? Tell me of times when you discussed these topics together or separately. ▪ What was most difficult or easy about this conversation? Was this typical? ▪ Who made a final decision about what you discussed? ▪ What are some of the reasons that you have not discussed these topics? ▪ What are some of the things you would like to discuss with your partner?
MPT interest	What are some of the reasons why you would want to use a dual‐purpose prevention product for pregnancy and HIV prevention? ▪ What would be your primary reason or motivation for using a dual prevention product—HIV prevention or pregnancy prevention? Explain. ▪ What are some of the things taking place in your life now or in the future that would influence your decision to use (or not to use) a DPP? ▪ What are reasons other couples may want to use a DPP?
	What are some reasons [you/you and your partner] would not want to use a dual‐purpose prevention product for pregnancy and HIV prevention? ▪ What could be happening in your lives that would make you less motivated or feel like you have less need/desire to prevent HIV and pregnancy? ▪ What are the reasons other couples may not want to use a DPP?
	If you wanted to prevent or space pregnancies and wanted to protect yourself from HIV, would you prefer to use one product that protects against both HIV and pregnancy, or would you rather use two different products—one for HIV prevention and another for pregnancy prevention? ▪ What would be better or worse about using a single product versus two separate products? ▪ What worries would you have about using a new medical product for both pregnancy and HIV prevention? ▪ How do you think a 2‐in‐1 product makes taking care of health easier or harder? ▪ What do you think are the different health effects of a single versus a dual‐purpose product? Both positive and negative effects.
MPT product preference	[*Participants were presented with a visual tool depicting product attributes and features to remind them of the choices of previous interview*.*]* Can you describe the product that [you/you and your partner] chose as your preference for the prevention of HIV and unintended pregnancies when you completed your DCE interview together? ▪ What are the things you like about the product you chose compared to the other possible products? ▪ What would you still change about the product that you chose and why? ▪ What concerns do you have about the product that you chose? ▪ Which other product would you choose, if your preferred product was not available, and why? ▪ What are the chances that you would use a dual‐purpose prevention product like the one you chose?
Partner and community influence	Could you describe how the choices you made individually versus together may have differed? ▪ Did you talk about your individual choices? ▪ What were the reasons why your choices were different? Why do you think they were aligned?
	How did you and your partner reach the decision to choose a particular product together? ▪ Which product features were the hardest to discuss or the ones that you disagreed on the most? Why? ▪ What product features did you not need to discuss? Why? ▪ What was one thing that your partner said that made you think differently about your choice or made you choose something different than what you initially would have chosen? ▪ How different would your preference have been if you had not chosen a product in your partner's presence? ▪ How important is it to make a choice like this together? Why is it important?
	What other factors may influence your preference for and interest in the future use of a dual‐purpose prevention product? ▪ What people or factors (e.g. cost, home environment, frequency of use, community rumours and perceptions, etc.)? ▪ To what extent may these have influenced your partner's choice of a DPP?

### 2.2 Data collection procedures

A semi‐structured interview guide was developed to explore couple dynamics and their influence on HIV prevention and contraceptive decisions, behaviours and preferences. The guide was developed by core study team members (JE, MH and AM) and then reviewed and revised for applicability and content validity by other protocol team members. Specific interview questions of interest for the present analysis pertained to decision‐making around existing and future HIV and pregnancy prevention options and attributes. We used neutral probes to gather detailed responses and elicit illustrative examples where necessary. This gave participants the chance to elaborate on information that they felt was important to tell the interviewer. Experienced study staff conducted the interviews in English and/or Luganda (in Uganda) and Shona (in Zimbabwe). Interviewers were trained on the semi‐structured interview guide, qualitative research principles and on issues specific to interviewing couples, such as handling conflict and engaging both members of the couple.

Interviewers were also trained to maintain some physical distance so as not to intrude when couples were completing activities. However, in cases where couples may not have been communicating verbally, interviewers prompted couples to verbalize their decision‐making processes to allow for accurate assessments of the couple's interaction. All interviewers in Zimbabwe were women, while participants in Uganda could choose a male or female interviewer. The staff member met participants at the study site for a 60‐minute, digitally recorded interview. The study staff used a transcription protocol to transcribe and translate interviews into English. Staff members who conducted the interviews then reviewed the transcripts for transcription and translation accuracy. All transcripts were de‐identified, underwent quality control checks and were stored on a HIPAA‐protected encrypted shared drive. The study was approved by Institutional Review Boards and Ethics Committees in Zimbabwe, Uganda, Canada and the United States.

### 2.3 Data analysis

Structured debriefing reports documenting key themes, questions from the semi‐structured interview guide and constructs from Lewis's model of interdependence and communal coping were used to derive the preliminary codebook [42]. Six team members (MK, SS, AM, MH, MS and AY) reviewed two randomly selected transcripts to identify and discuss additional codes. The study team reviewed the codebook for gaps and redundancies. Two co‐authors (MK and SS) then revised the codes and corresponding definitions to develop a final codebook. Four coders (MK, AY, SS and EO) independently applied codes to a total of 59 transcripts (19 joint and 40 individual interviews).

To achieve consistency in the application of codes, the study team (coders, content experts and site personnel) coded 8–10 transcripts per week and convened weekly to discuss discrepancies and reach a consensus. Members from each site's study team were present in the coding meetings to share insights based on their involvement in data collection and familiarity with transcripts. Throughout coding, the study team used consensus to resolve areas of disagreement, made necessary changes with code applications in Dedoose (SocioCultural Research Consultants, LLC; Los Angeles, California) and subsequently achieved satisfactory agreement to establish consistency in the application of codes.

Once all coding and inter‐rater reliability exercises were completed, the lead author developed an analysis plan. The analysis team used content analysis to examine the results of the coding process across participants and synthesized the information based on emerging themes and sub‐themes in summary memos. The analysis was structured to understand the differences and similarities between participants regarding dominance, couple versus individual decision‐making and conflicts in using existing HIV and pregnancy prevention products. Mentions of decision‐making changes in each interview were counted and summarized by direction of change and dominance (Table 2). The analysis team prepared detailed syntheses of findings from the summary memos and met every week to discuss key themes and ensure appropriate interpretation within the context of analytical objectives.

**Table 2:** Mentions of DCE decision change during the qualitative interview

	Total		Equal contribution		Male dominance		Female dominance	
	*N* = 59	(%)	*n* = 21	(%)	*n* = 20	(%)	*n* = 18	(%)
MP changed FP's decision	11	(19%)	3	(14%)	6	(30%)	2	(11%)
FP changed MP's decision	27	(46%)	10	(48%)	8	(40%)	9	(50%)
MP and FP same decision	5	(8%)	2	(10%)	1	(5%)	2	(11%)
No mention of decision change	16	(27%)	6	(29%)	5	(25%)	5	(28%)

## 3 RESULTS

### 3.1 Partner and couple characteristics

Our sample included 39 couples who participated in the MTN‐045 study. The median age of the female partner was 26 years (interquartile range [IQR] 18–38) and was 30 years for male partners (IQR 19–45). Most female partners had a secondary school education (64%, *N* = 25) and had previously given birth (74%, *N* = 29). Similarly, most male partners had a secondary school education (74%, *N* = 29) and were fathers (74%, *N* = 29).

The mean duration of relationships was 5.4 years (range 0.7–21) with most couples reporting that they jointly made decisions about family planning (74%, *N* = 29), were married or cohabitating (82%, *N* = 32) and currently used a pregnancy prevention method (82%, *N* = 32). The most used method was oral contraceptive pills (33%, *N* = 13) (Table 3). Across the 59 interviews, it was most often mentioned that female partners were able to change a male partner's DCE decision ( = 27, 46%) follow by no mention of decision change (*N* = 26, 27%), male partners changing a female partner's decision (*N* = 11, 19%) and both partners having the same decision (*N* = 5, 8%).

**Table 3:** Couple characteristics

	Total couples (*N* = 39)		Equal contribution (*N* = 19)		Male dominance (*N* = 10)		Female dominance (*N* = 10)	
Age in years (mean, median and range)								
Female partner	26, 25	(18–38)	26, 25	(18–38)	25, 23	(19–33)	25, 25	(18–38)
Male partner	30, 30	(19–45)	29, 30	(19–45)	31, 36	(20–40)	29, 28	(21–38)
Completed secondary school								
Female partner	25	(64%)	14	(74%)	5	(50%)	6	(60%)
Male partner	29	(74%)	14	(74%)	9	(90%)	6	(60%)
Married or cohabitating	32	(82%)	14	(74%)	10	(100%)	8	(80%)
Type of pregnancy prevention method currently used								
Oral pills	13	(33%)	6	(32%)	4	(40%)	3	(30%)
Injectable (or shot)	6	(15%)	4	(21%)	1	(10%)	1	(10%)
Implant	5	(13%)	4	(21%)	0	(0%)	1	(10%)
IUD	1	(3%)	0	(0%)	0	(0%)	1	(10%)
Male condom	10	(26%)	7	(37%)	2	(20%)	1	(10%)
Natural method	3	(8%)	3	(16%)	0	(0%)	0	(0%)
None	7	(18%)	1	(5%)	2	(20%)	4	(40%)
Relationship duration in years (mean, median and range)	5.4, 3	(0.7–21)	4.7, 2	(0.7–21)	6.1, 3.5	(2–14)	5.8, 2.5	(1–19)
Joint decision‐making on family planning	29	(74%)	16	(84%)	7	(70%)	6	(60%)
Interview type								
Joint	19	(49%)	12	(63%)	3	(30%)	4	(40%)
Separate	20	(51%)	7	(37%)	7	(70%)	6	(60%)

### 3.2 Decision‐making among existing HIV and pregnancy prevention options

In our sample, decision‐making discussions related to HIV prevention had different drivers and motivators than decision‐making discussions about pregnancy prevention. Decision‐making discussions related to HIV prevention centred on trust between partners and decision‐making discussions related to pregnancy prevention centred on attributes of contraceptive methods. The following paragraphs detail how HIV and pregnancy prevention decision‐making processes varied by relationship dominance (equal, male and female) but not by interview type (joint and individual).

Overall, levels of trust influenced frequency and topics of discussion as well as reported behaviours. For example, couples expressing a high level of trust reported increased HIV testing and limited condom use. In contrast, couples with a low level of trust reported infrequent HIV prevention discussions and constant vigilance which included asking the partner about where they go outside of the home, if they have met someone of the opposite gender at work and about who is calling them or sending them text messages. Couples with low levels of trust additionally reported that suggesting or accepting condom use indicated engagement in sexual relationships outside of the partnership.

MP: One only thing I caution her about is to be faithful. I told her that in case she is found HIV positive in future, that will be the end of our relationship. That if we are tested and she is found positive and I negative we shall end our relationship there and then. I know if she suggests condoms she is unfaithful. (Equal Dominance, Individual Interview, Zimbabwe)

Contraceptive method attribute discussions largely focused on differing partner preferences for return to fertility and interest in limiting the female partner's experience of side effects and decreases in the male partner's sexual pleasure. Among couples deemed to contribute equally to decision‐making (equal dominance), couples reported being open to communication on HIV and pregnancy prevention. Reasons for communication and prevention product use included needing to keep each other safe from HIV, birth spacing of future children and financial needs related to childcare.

MP: Usually it is her (female partner) who says that she does not at all want to get pregnant because of our financial status as I said earlier. She does not want to get into that before we are prepared. It is only that for the first pregnancy she was still young after school. And I also don't want it (getting another child) like I told you earlier. So we talk about it (prevention of pregnancy). She welcomed it and she said that the product will work for us if we sit down and choose together what can be better for us. (Equal Dominance, Individual Interview, Uganda)

In terms of HIV prevention, equal dominance couples reported testing for HIV more frequently, disclosing their results to their partners and deciding not to use condoms based on trust built from both behaviours. In terms of pregnancy prevention, equal dominance couples reported discussing the advantages and disadvantages of specific contraceptive methods together and choosing products that satisfied both partners and limited physical side effects for the female partner.

Couples dominated by either male or female member reported communication on HIV and pregnancy prevention to be infrequent and occasionally fraught. Reasons for infrequent communication included gendered beliefs on whether HIV and pregnancy prevention decisions should be openly discussed and assumptions that initiating discussions indicated the existence of other sexual partners.

MP: That when you are faithful to each other, you also become jealous of each other.

So, when talking about HIV prevention, you just emphasize one thing that you need to be faithful to each other to prevent HIV. But even if the woman is the one who is having other sexual partners, it is not easy for the man to tell the wife to use the condoms whenever she has sex with other men. It will appear as if he is giving her the permission to carry on with her reckless behaviour. So, that will stop people from discussing such issues. (Male Dominance, Individual Interview, Zimbabwe)

Male‐ and female‐dominated couples reported that fears of infidelity drove either partner to suggest HIV testing and condom use. However, condom use was largely dictated by male partners because condoms are regarded as a male product and male partners are often the final decision makers. There were differences in stated views regarding pregnancy prevention comparing male and female dominance couples. In male dominance couples, couples reported that pregnancy prevention choices were made according to the sexual needs and fertility interests of male partners. Male partners were unsupportive of methods with high costs, a delayed return to fertility or side effects that inhibited sex, such as irregular bleeding, decreased libido and vaginal dryness.

FP: The other issue is that most men do not like those family planning methods that we use because of side effects like irregular bleeding so they don't like them and that's why in most cases the men refuse the women to use them because you are bleeding and he cannot fulfil his need.

MP: And there is a way they change the woman

FP: That's when one says do not use it again

MP: There is a way they change the woman.

MP: The woman's sex libido is low and sometimes she doesn't completely want sex because she has no feelings

(Male Dominance, Joint Interview, Uganda)

In female dominance couples, couples reported that the female partner was “allowed” to choose the product because she was the one to use it and experience the side effects.

FP: He will say whatever you like. Sometimes I say I'm going for an injection and he just says, whatever you want is what you do. If what you have is not working for you, you can change it. So in the end it's my decision to make. (Female Dominance, Individual Interview, Zimbabwe)

### 3.3 Decision‐making regarding future MPT attribute preferences

When discussing decision‐making during the DCE activity, couples mentioned it as a negotiation among the prescribed set of potential product attributes with limited mentions of trust. Male partners were most concerned with side effects impacting sexual pleasure (change in menses and vaginal dryness), female partners were most concerned with side effects causing physical symptoms (headache, cramps and heavy bleeding) and both partners were concerned with the return to fertility.

FP: I think that at first, we were looking at my health as a woman, because I will be the one using the product, secondly we were looking at how it helps my partner to be free in the home [to be happy and satisfied sexually]. Such that I feel that if we use this product, his chances of having extra marital affairs will be slimmer, so it is important for us as family. I think that my partner was more concerned about how the vagina feels, that it should not be too dry, or too wet. Because dryness it will make me feel pain during sex, If it becomes too wet, it would not be good. (Male Dominance, Joint Interview, Uganda)

Many couples acknowledged that the attributes and product they chose when they completed the DCE alone was different than the attributes and product they chose when they completed the DCE with their partner.

MP: Haa, when I chose alone and when we made the choices together, what was different was, the woman was the one who would be using the products, so most of the choices were her preferences. Because in doing things we should be in agreement. Even if I have what I like, and my wife doesn't like it, I shouldn't oppose it. (Equal Contributor, Individual Interview, Zimbabwe)

When there was disagreement on the importance of specific attributes during the DCE, couples described a process of joint decision‐making where each partner was able to present their views and openly discuss their preferences. Though female partners across all dominance types were often able to convince their male partner to choose their preferred product or set of attributes (Table 2), the reasons for which their partner made the final decision varied by relationship dominance (equal, male and female). We did not find substantial differences by interview type (joint and individual).

Among equal and female dominance couples, couples often reported that the male partner “allowed” the female partner to make the decision on the final product, which was similar to reports from participants on existing product decision‐making. Reasons for relegating the final decision were that female partners cannot be forced and should like the product, are the ones who will use the product, experience side effects of the product and have a reliable judgement from their previous product experiences.

MP: Like I told you that my wife has a right to decide because the method is to go in her body, I could not impose anything on her, just because when it is time to go into her periods she informs me about it because I know what she goes through with the pain. So I realized that maybe that alone could save her the pain of the cramps. So I went by her choice as she had that fear that it may give her pains, though she had never used it. I would support her by advising her about side effects but since she experiences most of these I left her to make most of the final decisions. (Female Dominance, Individual Interview, Uganda)

Among male dominance couples, some female partners reported having to accept the choice of their male partner though they would have chosen differently if they could make an independent decision. In addition, some female partners in this couple dominance type reported not sharing their product preferences with their partners after discovering that they were different than the male partner's preferences. Reasons for accepting male partner preference included perceived fears of abandonment due to decreased sexual pleasure, male partners being the head of the household and limiting potential blame for outcomes.

FP: I accepted his choice. You know, even if it fails to work but when you had selected it together with your partner, there is no blaming you because you had agreed together with him. (Male Dominance, Individual Interview, Uganda)

### 3.4 Benefits of joint decision‐making during DCE

Reported benefits of joint decision‐making afforded by the DCE did not vary by dominance type or interview type. Benefits specifically included building trust, improving communication and deciding on an ideal product that served the couple instead of the individual. These benefits ultimately led to greater satisfaction in their final choice of products and attribute characteristics.

FP: [Making a choice as a couple] is important…we enjoyed it so much because we were together so no one would blame the other. We both understood the method and made a choice out of our decision. So in case of any changes no one will question the other because we are aware that there will either be a change or not. (Male Dominance, Joint Interview, Zimbabwe)

Couples reported that joint decision‐making during the DCE also increased male partner awareness of potential product side effects and how to support their partner in product use. Having discussions about all types of products also provided an opportunity to correct misinformation and learn more about existing options.

MP: I have no challenge sitting as a couple to make a decision. Actually, it is very important because if I make the choice alone there are some important things that I may ignore or I may not see it but if we are together she may advise me or I advise her so we have no challenges sitting as a couple. I learned more about products as they are now through the discussion and how to support her (Equal Contribution, Joint Interview, Uganda)

## 4 DISCUSSION

In a study of couples’ preferences for future MPT products to prevent HIV and unintended pregnancy conducted in Uganda and Zimbabwe, we collected qualitative data to understand how couples made decisions about the future MPT products presented, drawing also on discussions of their decisions related to existing HIV and pregnancy prevention options. We then evaluated whether decision‐making processes varied by couples’ relationship dominance (female‐dominated, male‐dominated and equal contribution) and interview type (couple interviewed jointly or separately). We found that decisions on products focused on trust and product attributes and varied by relationship dominance, but not by interview type.

Existing product decisions focused on trust and product attributes, while future product decisions exclusively focused on product attributes. Trust was primarily associated with the HIV prevention aspect of existing product decisions rather than the pregnancy prevention aspect. Trusting couples frequently communicated about HIV prevention and cooperatively made decisions on HIV testing and condom use. Mistrusting couples infrequently communicated about HIV prevention and reported that initiating related discussions or condom use signalled the existence of other sexual partners. Other studies among young couples in SSA have similarly found partners unable to discuss HIV testing and condom use without raising suspicions of infidelity which in turn impacted their ability to engage in HIV prevention behaviours [52, 53, 54, 55]. Given that the topic of trust was largely absent from future product decisions, our results suggest that MPTs could potentially minimize the need for continued HIV prevention negotiation among couples and the associated infidelity implications that prevent cooperative decision‐making to reduce HIV risk. In contrast to existing HIV prevention options, most couples in our sample reported open communication about existing pregnancy prevention options and a willingness to discuss future products. Thus, our results additionally suggest the potential acceptability of MPT communication among couples which will be important for uptake and adherence with future products.

Across existing and future products, preferences for product attributes varied by gender. Male partners were most concerned with limiting side effects impacting sexual pleasure (changes in menstrual bleeding and vaginal dryness), female partners were most concerned with limiting side effects causing physical symptoms (headaches, stomach pain and heavy bleeding) and both were concerned with the return to fertility. Though our results reflect known gender‐based barriers to contraceptive uptake [55, 56, 57], they add valuable context to the existing literature on MPT product preferences which has largely excluded the perspective of male partners [21, 58, 59, 60, 61, 62], who often wield considerable influence in couple‐based sexual and reproductive health decision‐making in the region [32, 39, 53, 63, 64, 65]. Our results also highlight the need for future MPTs to align with gendered product attribute preferences and the need for future MPT messaging to be clear about product side effects.

Across all relationship dominance types, couples reported making decisions together and female partners were often able to negotiate with male partners for their preferred product or set of product attributes. Many couples also acknowledged that the attributes and product they chose when they completed the DCE alone was different than the attributes and product they chose when they completed the DCE with their partner. Couples reported that the DCE provided a way to openly present their attribute preferences to their partner, learn about their partner's attribute preferences and make decisions that benefited the couple. Our results suggest that joint decision‐making activities (like the DCE) that focus on product attributes pertinent to choice and use could potentially provide an opportunity for a “transformation of motivation” (e.g. a mechanism in Lewis's model of interdependence and communal coping) which sets the stage for communal coping (e.g. MPT uptake and adherence). Female partners’ ability to negotiate for their preferences was also encouraging given prevailing gender norms and existing apprehension among researchers, practitioners and providers that involving male partners in sexual and reproductive decision‐making disempowers women [30, 55, 66, 67, 68, 69]. In addition, we know that many women do want to involve their male partners in their sexual and reproductive decision‐making, and doing so can improve the duration of product use [34, 39, 70, 71]. Future research to explore if couples’ attribute preferences and priorities elicited through the joint DCE align with their actual prevention choices will be essential to informing the development of provider‐delivered counselling and other interventions to support future MPT use.

This study has several important limitations. First, with a purposively selected sample, our findings cannot be generalized to a wider population. The couples in our study were willing to enrol in a study together and were interested in HIV prevention and family planning, which may not reflect the interests or experiences of other couples in Zimbabwe or Uganda. We were relatedly unable to measure the impact of relationship stability on MPT preference, which also limits our ability to generalize to the experiences of all couples in either country. Despite these limitations, we were able to include couples with a range of relationship dominance types and relationship lengths. Second, the transcripts were translated from Luganda and Shona to English and as with any translation, there was undoubtedly some level of information and understanding that was lost during the interpretation process. To minimize these losses, transcripts underwent quality control checks by those conducting and transcribing the interviews. Likewise, individuals from both site teams were involved in coding discussions and reviewing analysis. Third, it is possible that our results are partially reflective of observer presence during the DCE and social desirability bias underlying the reported interest in joint decision‐making. However, comments from male partners suggest that they felt comfortable describing their role as the primary decision maker. Separate interviews for male and female partners, with the option of gender‐matched interviewers, if preferred (Uganda only) allowed for openness. We did not find differences in patterns of decision‐making comparing the couple and individual interviews. Finally, though this was a couple‐based study, we must acknowledge that there are many other social network actors and processes that influence HIV and pregnancy prevention product use decisions.

## 5 CONCLUSIONS

Young women in many parts of SSA face high rates of HIV and unintended pregnancy and studies have documented keen interest by women in a product that combines HIV prevention and contraception. Given that MPTs will be used in the context of sexual relationships, ensuring uptake and adherence requires understanding whether and how to involve male partners in MPT use decision‐making for women who are in heterosexual relationships. The research activities in this study (DCE, survey questions completed jointly about preferences and qualitative interviews) prompted conversations that supported joint decision‐making among couples, regardless of relationship dominance type. These activities provided an opportunity for couples to openly present their attribute preferences to their partner, learn about their partner's attribute preferences, negotiate for their ideal set of attributes and ultimately make decisions that benefited the couple without disempowering the female partner. Developing couple‐based decision‐making aids or other tools may provide a mechanism through which women can negotiate with male partners to support their transformation of motivation, thereby encouraging MPT uptake and adherence when they become available for widespread use.

## COMPETING INTERESTS

The authors have no competing interests or financial interests relevant to this article to disclose.

## AUTHORS’ CONTRIBUTIONS

AMM and JE led study development and oversight as protocol chair and co‐chair. PM, JN, IR, PM and JE led data collection activities at their research sites and contributed to the interpretation of the results. MKSQ provided overall study coordination and contributed to study development and the interpretation of the results. MH helped conceptualize the analysis and contributed to the interpretation of the results. MCDS contributed to manuscript writing and the interpretation of the results. SRS coded and analysed data and contributed to manuscript writing and the interpretation of the results. MAL reviewed the manuscript and contributed to the interpretation of the results. JP contributed as DAIDS medical officer with key contributions to protocol development and management. NLB led manuscript development, analysis and writing. All authors approved the final manuscript as submitted and agree to be accountable for all aspects of the work.

## FUNDING

This research was supported by the Microbicide Trials Network (MTN). From 2006 until 2021, the MTN was part of the HIV/AIDS clinical trial network and was funded by the National Institute of Allergy and Infectious Diseases (UM1AI068633, UM1AI068615 and UM1AI106707), with co‐funding from the Eunice Kennedy Shriver National Institute of Child Health and Human Development and the National Institute of Mental Health, all components of the U.S. National Institutes of Health.

## DISCLAIMER

The content is solely the responsibility of the authors and does not necessarily represent the official views of the NIH.

## Data Availability

The data that support the findings of this study are available on request from the corresponding author. The data are not publicly available due to privacy or ethical restrictions.
